# Polyglobulia in patients with hemangioblastomas is related to tumor size but not to serum erythropoietin

**DOI:** 10.1186/s13053-018-0097-x

**Published:** 2018-09-11

**Authors:** Marie T. Krüger, Jan-Helge Klingler, Cordula Jilg, Christine Steiert, Stefan Zschiedrich, Vera Van Velthoven, Sven Gläsker

**Affiliations:** 1 Department of Neurosurgery, Freiburg University Medical Center, Freiburg, Germany; 2 Department of Urology, Freiburg University Medical Center, Freiburg, Germany; 3 Department of Internal Medicine, Section for Preventive Medicine, Freiburg University Medical Center, Freiburg, Germany; 4 Department of Neurosurgery, Universitair Ziekenhuis Brussel, Laarbeeklaan 101, 1090 Brussel, Belgium

## Abstract

**Background:**

Hemangioblastomas are associated with elevated hemoglobin (Hb) levels (polyglobulia), which is associated with a higher risk for cerebral stroke, cardiac infarction and pulmonary embolism. The pathomechanism of polyglobulia remains unclear and different theories have been postulated. Among those are elevated serum erythropoietin (EPO) levels caused by secretion of the tumor or associated tumor cyst.

**Methods:**

To elucidate the pathomechanism, we systematically investigated the relation between polyglobulia, serum EPO level, size of the solid tumor and associated cyst in hemangioblastomas. We prospectively evaluated hemoglobin and EPO levels in a series of 33 consecutive patients operated on hemangioblastomas in our center. We measured the size of the solid tumor and associated cyst in magnetic resonance imaging. Statistical evaluations were performed using the Fisher’s exact test and student’s t-test.

**Results:**

As a result five patients had elevated hemoglobin levels. Only one of these had an elevated serum EPO level. Of 26 patients with normal hemoglobin levels, 4 patients had elevated EPO levels.

Patients with low or normal hemoglobin levels (84%) had an average tumor size of 0.8 cm^3^, which differed significantly from patients with elevated hemoglobin levels (16%), who had an average solid tumor size of 8.0 cm^3^ (*p* < 0.05). We did not observe a significant correlation between EPO levels or polyglobulia and associated cysts.

**Conclusions:**

We therefore conclude that in contrast to previous case reports and interpretations, our data show no correlation between polyglobulia and EPO levels or associated cysts in patients with hemangioblastomas. In fact, it is the size of the solid tumor that correlates with polyglobulia.

The study was retrospectively registered in the German Clinical Trial Registry on 10 July 2014; Trial registration: DRKS00006310.

## Background

Hemangioblastomas are highly vascular neoplasms, which can occur as sporadic tumors or as part of the von Hippel–Lindau (VHL) disease [1]. They comprise approximately 3% of all tumors of the central nervous system and are mainly located in the cerebellum and spinal cord [2]. The tumors are composed of a dense reactive angiogenetic capillary network with “stromal” cells in between, which are the neoplastic component. These “stromal” cells represent developmentally arrested hemangioblast progenitor cells with hematopoietic and vasculogenic differentiation potential [3–5].

Polyglobulia is defined as pathologically elevated hemoglobin (Hb) level in the blood of affected patients. It results in a higher risk to develop thrombosis, which can lead to severe complications such as cerebral stroke, cardiac infarction and pulmonary embolism. An association of hemangioblastomas and polyglobulia has been frequently reported in single case reports [6–9] and small series [10, 11]. In the past, however, polyglobulia has been reported more frequently with incidences up to 49% [11]. We and others have more recently assessed the incidence much lower with numbers around 5–8% [12].

So far, causative mechanisms have remained elusive. Many different possible mechanisms have been discussed. Among these are extramedullary hematopoiesis [5] within the tumors as well as secretion of erythropoietin (EPO) or other hematopoietic factors by the tumor itself or its associated cysts [5, 6, 8, 13, 14]. Furthermore certain types of VHL mutations such as loss-of-functions mutation VHL^R200W^ that causes Chuvash polycythemia [15, 16] or other VHL-associated tumors such as clear cell renal carcinoma or pheochromocytomas have been discussed.

We have recently postulated that the tumor itself causes polyglobulia since patients are cured from polyglobulia after its removal [2]. Hemangioblastomas with polyglobulia were often described as large and solid [1, 11, 12, 17]. A causative correlation, however, has not yet been investigated.

To elucidate the correlations of possible causative factors such as EPO levels, tumor size and cystic formations, co-morbidities and VHL mutation specificity in patients with hemangioblastomas, we systematically investigated a consecutive series of patients operated on hemangioblastomas in our department.

## Methods

### Patients

Our hospital serves as reference center for patients with hemangioblastomas and VHL disease. For this retrospective study, preoperative EPO and Hb concentrations were measured in blood of a consecutive series of patients with central nervous system hemangioblastomas operated in our center between 01/2009 and 12/2012. We also measured the size of the solid part of the tumors and associated cyst. We collected data including patients’ age, sex and medical history in regard to factors that could influence Hb or EPO levels. Among those were smoking, medications, anemia, hypoxia related diseases (chronic obstructive pulmonary disease (COPD), sleep apnea) and exposure to altitude. Patients suffering from these diseases were excluded from the study. VHL-associated manifestations such as pheochromocytoma, renal or pancreatic tumors or cysts were also registered.

The ethics committee of the University of Freiburg approved the study. All patients gave informed consent.

### Hb and EPO levels

Hb levels were considered normal from 12.0–15.0 g/dl in female and 13.6–17.2 g/dl in male [7]. EPO levels were considered normal from 3.7–31.5 g/dl in female and male.

### Measurement of solid tumors and associated cysts

**Fig. 1:**
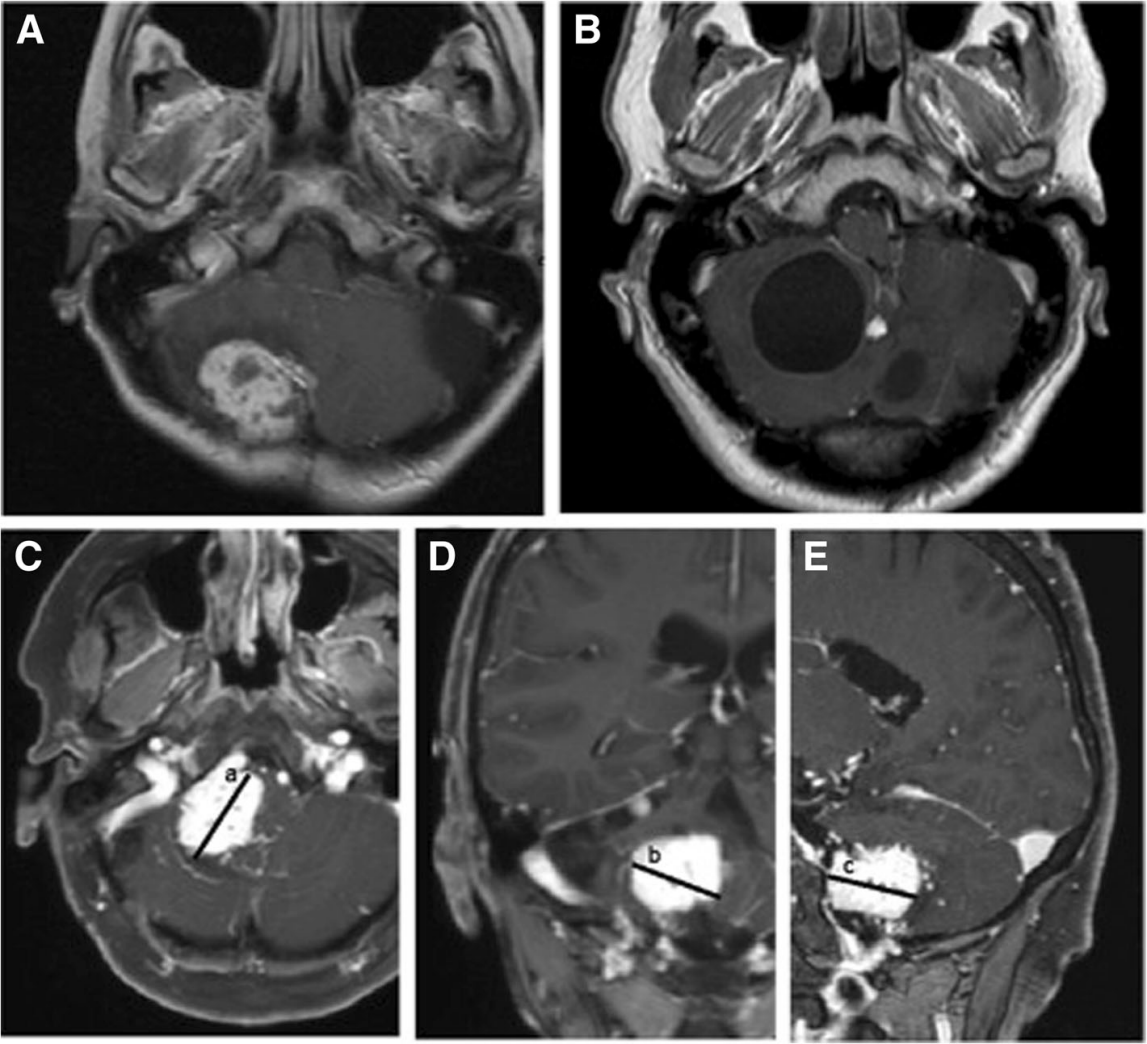
Examples of hemangioblastomas and measurement of solid tumor size in MRI; MRI scans with example of (**a**) solid tumor and (**b**) associated tumor cyst. Example of measurement of solid tumor size in contrast-enhanced MRI (**c**) axial, (**d**) coronar and (**e**) sagittal. Values a, b, c were multiplied and divided by 2 to receive tumor size volume in cm^3^

Size of the solid tumor and associated cysts was measured in contrast-enhanced magnetic resonance imaging (MRI) scans. The size of the solid tumor (contrast-enhanced region) or associated cyst (contrast-free region) was measured in three dimensions (height, length and width) (Fig. 1) and divided by 2. Only tumors larger than (0.5 × 0.5 × 0.5)/2 cm (0.1 cm^3^) were registered. If patients had multiple tumors, only the largest tumor was recorded.

### Statistical analyses

Descriptive statistics was done by calculating means, medians and standard deviations. To analyze predictors for polyglubulia, univariate and multivariate binary logistic-regression was performed. Continuous variables were compared with a two-sided unpaired Student’s t-test. Fisher’s exact test was used for analyzing contingency tables. The level of significance was indicated by a *p*-value< 0.05. All statistics were done with SPSS v19 (IBM Corp. Armonk, NY, USA).

### Molecular genetic analyses

All patients were investigated for germline mutations of the *VHL* tumor suppressor gene. Molecular genetic analyses were performed as previously described [7, 15].

## Results

### Patients

Thiry-three patients were primarily included in the consecutive series. Two had to be excluded from the study due to co-morbidities that could influence Hb or EPO levels. One female patient suffered preoperatively from anemia, one male patient suffered from COPD. From the 31 included patients, 16 patients were female (52%) and 15 (48%) were male (Table 1). The patients’ age ranged from 16 to 75 years with an average of 43.6 years. 64.3% of patients either had renal cell carcinomas or cysts, 53.6% had pancreatic tumors or cysts and 14.3% had pheochromocytomas. The distribution of renal cell carcinomas among patients with polyglobulia or without was not significantly different (*p* = 1.0). 9/26 (35%) patients without polyglobulia and 1/5 (20%) patients with polyglobulia had concomitant renal cell carcinomas (Table 2).

**Table 1:** Patients characteristics from 31 individuals

Pat	Sex	Age	EPO, mU/ml	EPO level	Hb, g/dl	Hb level	VHL mutation	Cyst	Size, cm3
1	f	57	24	n	12.9	n	+	+	0.1
2	f	60	3	↓	14	n	+	–	8.1
3	f	42	41	↑	11.9	↓	746 T/A	–	0.9
4	f	39	34.2	↑	14.7	n	+	–	2.2
5	f	48	13.2	n	13.7	n	del 2b	+	0.3
6	f	55	7.6	n	16.6	↑	407 C/T	+	9.6
7	f	37	15.5	n	12.2	n	+	+	0.1
8	f	57	14.9	n	14.8	n	505 T/C	+	0.1
9	f	20	11.5	n	14.2	n	+	+	0.1
10	f	55	7.3	n	14.1	n	+	+	0.1
11	f	42	22	n	11	↓	no	+	0.4
12	f	35	20.5	n	14	n	+	+	1.4
13	f	44	13.2	n	13.3	n	+	–	0.8
14	f	53	7.9	n	15.8	↑	+	–	5.1
15	f	75	36.3	↑	17.1	↑	505 T/C	+	13.6
16	f	56	12.9	n	13.7	n	505 T/C	–	0.4
17	m	47	17.5	n	18.3	↑	694 C/T	–	5.3
18	m	39	5.6	n	16.1	n	407 C/G	+	0.2
19	m	39	8.8	n	15.7	n	del ex 2	+	0.1
20	m	39	10.2	n	14.9	n	del ex 3	–	0.1
21	m	52	9.6	n	15.5	n	no	+	0.1
22	m	28	11	n	13.6	n	430 C/T	+	0.1
23	m	27	11.5	n	14	n	del 5 kb	+	0.4
24	m	44	106	↑	15.1	n	del ex1–3 IRAK2	+	0.1
25	m	52	14.5	n	14.6	n	738 C/A	–	1.6
26	m	41	7.6	n	19.4	↑	553 G/A	–	6.7
27	m	25	15.1	n	16.3	n	+	+	0.1
28	m	31	10.6	n	16	n	del 2 kb	–	0.1
29	m	39	7.3	n	16.6	n	676 + 2 T/C	+	1.3
30	m	48	21.6	n	14.8	n	677-1G/C	–	0.2
31	m	32	87.6	↑	15.1	n	454 C/T	–	0.2

**Table 2:** Binary logistic regression model predicting polyglobulia in 31 individuals

Variable	Univariate OR (95% CI); P	Multivariate OR (95% CI); P
Tumour volume, cm3	2.275 (1.21–4.28); 0.011	2.221 (1.04–4.74); 0.039
Presence of associated cyst Cyst versus no cyst	0.489 (0.06–3.44); 0.472	0.166 (0.01–54.7); 0.544
EPO level, mU/ml	0.982 (0.91–1.05); 0.602	0.987 (0.81–1.19); 0.892
VHL mutation		
Non-truncating versus truncating	7.180 E8 (0.00–0.00); 0.999	5.4 E7 (0.00–0.00); 0.999
Miscellaneous versus truncating	1.469 E8 (0.00–0.00); 0.999	7.1 E6 (0.00–0.00); 0.999
Concomitant renal cell carcinoma (RCC) RCC versus no RCC	0.472 (0.05–4.88); 0.529	2.790 (0.01–436.9); 0.691

Table shows results for binary logistic regression model predicting polyglobulia in 31 individuals for selected variables: tumor volume, presence of cyst, erythropoietin (EPO) level, VHL mutation (truncating versus non-truncating) and renal cell carcinoma (RCC). Tumor volume was a significant parameter predicting polyglobulia. OR indicates Odds ratio; P, *p*-value and 95% CI, 95% confidence interval

There was no statistically significant association for polyglobulia and other VHL-associated diseases such as renal cysts (*p* = 1.0), pancreatic tumors (*p* = 1.0) or pheochromocytomas (*p* = 0.12), only pancreatic cysts showed a significant association (*p* = 0.04). None of the patients was a heavy smoker (> 20 cigarettes a day), took medication that influences Hb or EPO levels or had been exposed to altitude within 1 month before measurements.

### Hb and EPO levels

**Fig. 2:**
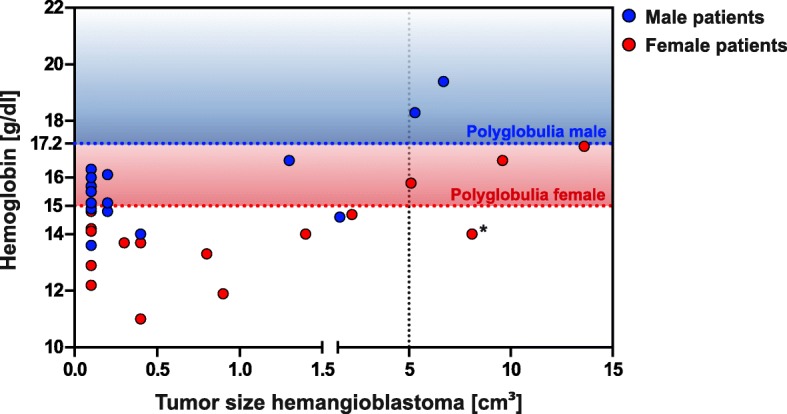
Distribution of hemoglobin levels and tumor size; Red line marks level for high values in female, blue line marks level for high values in male. Except for 1 value (*), elevated hemoglobin levels were only found in patients with tumor size larger than 5 cm^3^

Hb levels ranged from 11.0 to 19.4 g/dl with an average of 14.4 g/dl (15.7 g/dl in male and 14.0 g/dl in female) (Fig. 2). EPO levels ranged from 3.0 to 106.0 mU/ml with an average of 20.3 mU/ml (22.9 mU/ml in male and 17.8 mU/ml in female). Patients showing polyglobulia (*n* = 5) had no significant different EPO-level (mean 5.38 mU/ml) compared to patients without polyglobulia (21.25 mU/ml; *p* = 0.60; CI: -28.52 to 16.78).

Table shows 31 patients with normal (n), elevated (↑) or low (↓) hemoglobin (Hb) and erythropoietin (EPO) levels. Sex and age of patients, as well as type of von Hippel-Lindau (VHL) mutation, associated cyst and solid tumor size are stated. Elevated values were (> 17.3 g/dl in male or > 15.1 g/dl in female) measured preoperatively in blood serum. Size is stated in cm3 for largest hemangioblastoma. Large hemangioblastomas (> 5 cm3) are marked bold.

Pat indicates patient number; f, female; m, male; and +, undefined VHL mutation.

### Associated tumor cysts

Seventeen of the 31 patients carried an associated tumor cyst (7 male and 10 female). These were 15 (58%) of the 26 patients with normal Hb levels and two (40%) of the 5 patients with elevated Hb levels. Neither by performing univariate nor by multivariate binary logistic regression, the presence of an associated tumor cyst was a predictive variable for polyglobulia (Table 2).

### Molecular genetic analysis

Molecular genetic investigation of 31 patients with hemangioblastomas revealed 29 patients (94%) with *VHL* germline mutation. Patients without mutations did not exhibit clinical stigmata of VHL disease. The over-representation of VHL patients in our population results from referral bias since our center is acting as a reference center for VHL disease. Of the two patients without *VHL* mutation, one was male (patient no. 21) and had normal Hb and EPO levels, the other patient was female (patient no. 11) and had low Hb and normal EPO levels. Of the other 29 patients included in our study, *VHL* mutations were distributed without clustering throughout all 3 exons of the *VHL* tumor suppressor gene. In 10 patients no exact location was determined. In the other 19 patients non-truncating mutations were found in 13 of 19 patients (68%) and truncating mutations in 6 of 19 patients (32%). There was no significant influence of the molecular genetic of *VHL* mutations (truncating versus non-truncating) on the presence or absence of polyglobulia (Table 2).

### Size of solid tumors and Hb levels

Regarding the size of the solid tumors of all 31 individuals mean tumor volume was 1.93 cm^3^ (median: 0.30 cm^3^, SD: 3.30 cm^3^). Patients with low or normal Hb levels (26/31, 84%) had an average tumor size of 0.70 cm^3^, which differed significantly from patients with elevated Hb levels (4/31, 16%), who had an average solid tumor size of 8.0 cm^3^ (*p* < 0.0001; CI: 5.314 to 9.299) (Fig. 2). Furthermore tumor volume turned out to be the only predictive variable in univariate and multivariate regression model with respect to polyglobulia (Table 2).

By performing univariate binary logistic regression only tumor volume was a significant parameter predicting polyglobulia (OR: 2.275; *p* = 0.011; CI: 1.21–4.28). Neither presence of RCC, EPO-level, associated tumor cysts nor VHL-mutation (truncating versus non-truncating) had any significant impact in predicting polyglobulia (Table 2) By performing multivariate binary regression again only tumor volume was a robust independent predictor for polyglobulia. The remaining variables had no significant impact (Table 2).

## Discussion

Hemangioblastomas were found to be associated with polyglobulia, however, frequencies described in literature vary profoundly and the causative mechanisms have remained elusive. We consistently found that removal of the large hemangioblastomas in patients with polyglobulia led to normalization of Hb levels, which is in line with prior report [6, 8–10, 14, 18].

Many suggestions for causative mechanisms have been discussed. Among those are extramedullary hematopoiesis [5] or secretion of EPO by the tumor itself [6, 10, 14] or its associated cysts as well as genetic mutations of the *VHL* gene [15, 16]. Moreover VHL-associated tumors such as renal cell carcinomas were described to be causative for elevated Hb or EPO levels.

In this work we systematically investigated those mechanisms most commonly discussed in literature.

### Polyglobulia and EPO levels and tumor cysts

One mechanism most often suggested is secretion of EPO by the tumors. This glycoprotein influences Hb levels by regulating the production of erythrocytes and was frequently detected in cyst fluid of hemangioblastomas. Significant elevated EPO levels in the blood serum have often been reported [6–10]. On the contrary, other case reports described normal EPO levels in the serum of patients with polyglobulia [14, 19, 20].

In our study, which is the largest to date, only one of five patients with elevated Hb had elevated EPO levels. The other patients had normal EPO levels. We therefore conclude that the occurrence of polyglobulia in patients with hemangioblastomas does not depend on serum EPO levels. We did also not find an association of elevated EPO levels and tumor cysts. The exact mechanism and function of EPO that was found in cysts and blood serum is therefore still not known. Jeffreys and co-workers^13^ suggested that a significant release of the erythropoietic stimulation factor from the cyst fluid into the blood could be just an ‘uncommon occurrence’. Considering that hemangioblastomas represent embryonic cells, the produced EPO might be a form of fetal EPO, which is measurable but not functional like its adult form.

### Polygobulia is not associated with specific VHL mutations or other VHL associated tumors

Certain types of *VHL* mutations such as loss-of-functions mutation VHL^R200W^ that causes Chuvash polycythemia were described to be causative for poylglobulia.

In this study we did not detect any correlation between polyglobulia and certain *VHL* germline mutations. The distribution of germline mutations corresponded to the known distribution of all VHL patients in South Germany [18].

Patients with polyglobulia and VHL disease often carry associated comorbidities such as renal or pancreatic cysts or tumors as well as pheochromocytomas. In our study we did not find a relevant correlation between patients with other VHL associated tumors and polyglobulia.

### Polyglobulia and tumor size

In this study we found a significant association of polyglobulia and tumor size. Interestingly, the literature of the distant past stated incidences for polyglobulia much higher than in more recent studies [2]. In 1972, Palmer [11] published a case series of 81 patients with hemangioblastomas stating that 25 patients (49%) of the patients with available Hb levels (51 cases) suffered from polyglobulia. In 1958, Starr and co-workers [21] had reported on incidences of elevated Hb levels of 36% (11% with pathological levels) in a series of 106 patients.

Hemangioblastomas with polyglobulia were often described as large and solid [1, 11, 12, 17]. A causative correlation, however, has not yet been investigated.

The increased incidence in older reports might be attributable to lower sensitivity of former radiographics leading to detection of tumors at a later stage and therefore bigger size. Before 1986, ventriculography was the primary imaging method and was then superseded by the computed tomography [12]. Nowadays annual MRI scans of the neuroaxis are performed in VHL patients and therefore hemangioblastomas are detected at much smaller stages.

Moreover, many patients died shortly after brain surgery in the distant past. In 1957, Jeffreys et al. [13] described a series of 7 patients with hemangioblastomas. Two of them died within the first 2 days after the operation, only 2 survived more than 9 months. Therefore, it was not common to operate on patients who only had mild or no symptoms. At that time, hence, the high risk of operation, late radiographic diagnosis of hemangioblastomas and high incidence of polyglobulia may have led to surgical resection merely of hemangioblastomas that have already reached a larger tumor size.

In several publications, the exact tumor size was missing. If noted, large solid tumors were dominant [11, 17]. Carpenter et al. [17],^3^ for example, published one of the first case reports on this topic in 1943 describing two cases. One patient was a 52 year-old male with severe neurological symptoms who had a preoperative Hb level of 23.0 g/dl. His tumor was described as solid, measuring (3.5 × 3.5 × 2.5)/2 cm (15 cm^3^). Three months after resection of the tumor, Hb level had dropped to a normal value of 14.0 g/dl. The other patients was a 29 year-old male with only mild symptoms whose tumor was solid, measuring (3 × 3.5 × 2.5)/2 cm (13.1 cm^3^) Preoperative Hb level of 20.0 g/dl dropped down to 14.7 g/dl after resection of the tumor.

Nowadays hemangioblastomas are diagnosed at much earlier stages and the growth of single lesions can be registered much faster. It is now generally known that the outcome for patients with hemangioblastomas which are operated before neurological deficits occur are better than in those patients operated at later stages [22]. Hemangioblastomas are therefore operated earlier and, hence, at much smaller sizes. In conclusion this would explain why today hemangioblastomas do not grow that large and therefore do not cause polyglobulia as frequently as in the past.

## Conclusion

This is the first systematic investigation of possible mechanisms causative for polyglobulia in patients with hemangioblastomas. In contrast to previous case reports and interpretations, our data show no association between polyglobulia and EPO levels or concomitant cysts in patients with hemangioblastomas. We could demonstrate that tumor volume was the only independent factor predicting polyglobulia. Lower incidences of polyglobulia in current studies compared to the past may be attributed to improved radiographic techniques and clinical screening programs leading to earlier detection and treatment of hemangioblastomas at a smaller size. Further investigations especially of molecular mechanisms of the solid tumors are necessary to identify causative parameters for polyglobulia leading to better understanding and potentially incorporating novel diagnostic tools or treatments into clinical practice.

## Abbreviations

95% CI: 95% confidence interval
COPD: 9chronic obstructive pulmonary disease
EPO: Erythropoietin
Hb: Hemoglobin
MRI: Magnet resonance imaging
OR: Odds ratio
P: *p*-value
RCC: Renal cell carcinoma
VHL: Von Hippel–Lindau
